# Pilates for Overweight or Obesity: A Meta-Analysis

**DOI:** 10.3389/fphys.2021.643455

**Published:** 2021-03-11

**Authors:** Yi Wang, Zehua Chen, Zugui Wu, Xiangling Ye, Xuemeng Xu

**Affiliations:** ^1^The Fifth Clinical Medical College of Guangzhou University of Chinese Medicine, Guangzhou, China; ^2^Guangdong Second Traditional Chinese Medicine Hospital, Guangzhou, China

**Keywords:** Pilates, overweight, obesity, review, meta-analysis

## Abstract

**Background:** Evidence for the efficacy of Pilates for the modulation of body weight and body composition is unclear.

**Objective:** This meta-analysis aimed to evaluate the effects of Pilates on body weight and body composition in adults with overweight or obesity.

**Data Sources:** The PubMed, Cochrane Library, Web of Science, China National Knowledge Infrastructure (CNKI), and EMBASE databases were systematically searched from the inception dates to 12 November 2020 for relevant randomized controlled trials (RCTs).

**Study Selection:** Randomized controlled trials comparing Pilates with other physical exercises or without any intervention were included.

**Data Extraction and Synthesis:** Three reviewers independently performed the data extraction and assessed study quality. The mean differences (MDs) and 95% confidence intervals (CIs) for pooled data were calculated.

**Main Outcomes and Measures:** Outcome measures were body weight, body mass index (BMI), body fat percentage, lean body mass, and waist circumference.

**Results:** Eleven RCTs with 393 subjects were included. This study revealed that Pilates dramatically reduces body weight (MD = −2.40, 95% CI: [−4.04, −0.77], *P* = 0.004, *I*^2^ = 51%), BMI (MD = −1.17, 95% CI: [−1.85, −0.50], *P* = 0.0006, *I*^2^ = 61%), and body fat percentage (MD = −4.22, 95% CI: [−6.44, −2.01], *P* = 0.0002, *I*^2^ = 88%) in adults with overweight or obesity. The reduction in body weight and body fat percentage appears to be more pronounced in studies including participants with obesity only, and the efficacy of Pilates for the improvement of body weight and BMI appears to be more evident in longer intervention duration. However, Pilates has no significant effect on waist circumference (MD = −2.65, 95% CI: [−6.84, 1.55], *P* = 0.22, *I*^2^ = 0%) and lean body mass (MD = −0.00, 95% CI: [−1.40, 1.40], *P* = 1.00, *I*^2^ = 23%).

**Conclusions:** Pilates dramatically reduces body weight, BMI, and body fat percentage in adults with overweight or obesity. Large-scale and well-designed RCTs with improved methodology and reporting are urgently needed to further confirm these results.

## Introduction

Overweight and obesity, which are defined as abnormal or excessive body fat accumulation (World Health Organization, 2020), are the leading variable risk factors for chronic diseases and premature death (World Health Organization, 2018). According to the body mass index (BMI) (kg/m^2^), for adults, overweight is a BMI ≥ 25, whereas obesity is a BMI ≥ 30 (World Health Organization, 2020). Statistically, in 2016, 39% of adults aged 18 years and over were overweight, and 13% were obese; the prevalence of overweight and obesity among children and adolescents aged 5–19 was about 18%. Thirty-eight million children under the age of 5 were overweight or obese in 2019 (World Health Organization, 2020). Overweight and obesity have considerable negative socioeconomic impacts due to the high prevalence (Hammond and Levine, 2010). Besides excessive caloric intake, a sedentary lifestyle is also a major factor leading to overweight and obesity (Morgen and Sørensen, 2014). Therefore, regular physical activity has been recommended as one of the most effective prevention and treatment options for people with overweight or obesity (Expert Panel Members et al., 2014; World Health Organization, 2020). Given that some common physical exercises are ineffective in losing weight, and even may damage the musculoskeletal system due to knee joints overload in individuals with overweight or obesity (Girard et al., 2017; Hun-Young et al., 2019), it is necessary to investigate the alternative forms of exercise for treating overweight and obesity.

One such alternative modality of exercise that is widely used for health enhancement and adjuvant treatment in various diseases is Pilates (Sharma et al., 2018; Eliks et al., 2019; Fernández-Rodríguez et al., 2019). Pilates exercise, which was originated in the 1920s, mainly involved isometric contractions of the core muscles (Chen Z. et al., 2020). Moreover, the Pilates exercise system combined practical movement styles and ideas of martial arts, dance, gymnastics, and yoga with philosophical notions and followed six basic principles of centering, concentration, control, precision, flowing movements, and breathing (Latey, 2001). Pilates, although not designed to reduce body weight, can be considered a good option for people with overweight or obesity who have difficulty in adhering to those monotonous traditional physical exercises (Vancini et al., 2017; de Souza Cavina et al., 2020). Furthermore, Pilates does not require high costs to practice and produces high concentration in the muscle core, which could contribute to the implementation of this exercise method in clinical practice to improve body weight and body composition (de Souza Cavina et al., 2020). While the overall effects of physical exercises on body weight in people with overweight or obesity are now well-known, Pilates is not. Despite several studies have been conducted to assess the physical effects of Pilates for individuals with overweight or obesity, the results have been diverse with no clear consensus (Hagner-Derengowska et al., 2015; Rayes et al., 2019; Jung et al., 2020). Additionally, there is no systematic review or meta-analysis on Pilates for treating overweight and obesity to date. Therefore, the aim of this systematic review and meta-analysis of randomized controlled trials (RCTs) was to evaluate the effects of Pilates on body weight and body composition in adults with overweight or obesity.

## Methods

### Search Strategy

This review was conducted in accordance with the Preferred Reporting Items for Systematic Reviews and Meta-Analyses (PRISMA) guidelines (Moher et al., 2009). PubMed, the Cochrane Library, Web of Science, the China National Knowledge Infrastructure (CNKI), and EMBASE databases were systematically searched from the inception dates to 12 November 2020. We applied no language restrictions. We used the following combined text and MeSH terms: “Pilates,” “overweight and obesity,” and “Randomized Controlled Trial.” The complete search used for PubMed was: ((((((((((((((“Overweight” [Mesh]) OR (“Obesity” [Mesh])) OR (Overweight [Title/Abstract])) OR (Obesity [Title/Abstract])) OR (adipose tissue hyperplasia [Title/Abstract])) OR (adipositas [Title/Abstract])) OR (adiposity [Title/Abstract])) OR (alimentary obesity [Title/Abstract])) OR (body weight, excess [Title/Abstract])) OR (corpulency [Title/Abstract])) OR (fat overload syndrome [Title/Abstract])) OR (nutritional obesity [Title/Abstract])) OR (obesitas [Title/Abstract])) AND (((((((((“Exercise Movement Techniques” [Mesh]) OR (Movement Techniques, Exercise [Title/Abstract])) OR (Exercise Movement Technics [Title/Abstract])) OR (Pilates-Based Exercises [Title/Abstract])) OR (Exercises, Pilates-Based [Title/Abstract])) OR (Pilates Based Exercises [Title/Abstract])) OR (Pilates Training [Title/Abstract])) OR (Training, Pilates [Title/Abstract])) OR (Pilates [Title/Abstract]))) AND ((randomized controlled trial[pt] OR controlled clinical trial[pt] OR randomized[tiab] OR placebo[tiab] OR drug therapy[sh] OR randomly[tiab] OR trial[tiab] OR groups[tiab] NOT (animals [mh] NOT humans [mh]))). Additionally, the reference lists of all retrieved articles and relevant reviews were manually screened for potentially eligible studies. Two reviewers (Y Wang and ZH Chen) independently screened and selected papers, a third reviewer (ZG Wu) was consulted to resolve disagreements. The search strategy is detailed in Supplementary Table 1.

### Selection Criteria

We formulated the study's eligibility criteria using the participants, interventions, comparison, outcomes, and study design (PICOS) description model (Guyatt et al., 2011).

#### Participants

Inclusion criteria:

- Adult subjects aged 18 or over with overweight or obesity (overweight, BMI ≥25.0 kg/m^2^; obesity, BMI ≥30.0 kg/m^2^)

Exclusion criteria:

- Pregnancy
- Participants with any contraindication of exercise therapy
- Participants with eating disorders

#### Intervention

- Pilates

No restrictions were made in terms of the type of Pilates, the duration, dose, or intensity of the intervention. Furthermore, studies with dietary intervention were excluded.

#### Comparators

- Pilates vs. other physical exercises
- Pilates vs. without any intervention

#### Outcomes

The included studies had to assess at least one primary outcome related to body weight or body composition:

- Body weight
- Body mass index
- Body fat percentage
- Lean body mass
- Waist circumference

In addition, the safety of Pilates intervention was considered secondary outcomes.

#### Study Design

- RCTs
- Published in English or Chinese language

### Data Extraction

Two reviewers (Y Wang and ZH Chen) independently extracted the following data from the included studies: participant characteristics (e.g., age, gender, year), study characteristics (e.g., author names, publication year, region, study design, intervention type, intervention characteristics, sample size), and related study outcomes. Any disputes were resolved by discussing with a third reviewer (ZG Wu). If necessary, the corresponding authors were contacted for additional information.

### Quality Assessment

The methodological quality of included studies was assessed using the Physiotherapy Evidence Database (PEDro) scale (Maher et al., 2003; Macedo et al., 2010), which is used to assess the quality of RCTs with physical therapist interventions. The PEDro scale includes 10 items on random allocation, concealed allocation, similarly at baseline, subjects blinding, therapists blinding, assessors blinding, dropout rate <15%, intention to treat analysis, between-group statistical analysis, and point and variability measures; the score ranges from 0 to 10. A score ≥7 was considered “high quality,” a score of 5 or 6 was considered “moderate quality,” and ≤4 “poor quality” (de Souza Cavina et al., 2020). Moreover, the quality of evidence was assessed using the Grading of Recommendations Assessment, Development, and Evaluation System (GRADEprofiler, version 3.6; GRADE Working Group, 2004; Higgins et al., 2020). The assessment process was also conducted by two independent reviewers (Y Wang and ZH Chen), and the consensus approach was once again adopted.

### Statistical Analysis

Review Manager (version 5.3) and Stata (version 13.0) were used to conduct statistical analyses. Data were presented as mean ± standard deviation (SD) or 95% confidence interval (CI). All continuous variables were pooled by mean difference (MD) or MD with 95% CI. Heterogeneity was assessed by Higgins *I*^2^ statistic and 0–25% suggests very low heterogeneity, 25–50% low heterogeneity, 50–75% moderate heterogeneity, and more than 75% high heterogeneity (Higgins and Thompson, 2002; Higgins et al., 2020). The fixed effect models would be enabled if *I*^2^ < 50%; otherwise, a random-effect model was applied. To explore potential sources of heterogeneity between studies, subgroup and meta-regression analyses were conducted based on intervention duration and participant type. Sensitivity analyses were used to test the robustness of significant results. In addition, the visual inspection of funnel plots and statistical asymmetry tests (Begg's and Egger's tests) were used to assess publication bias. Statistical significance was considered for *P* < 0.05.

## Results

### Study Selection

**Figure 1:**
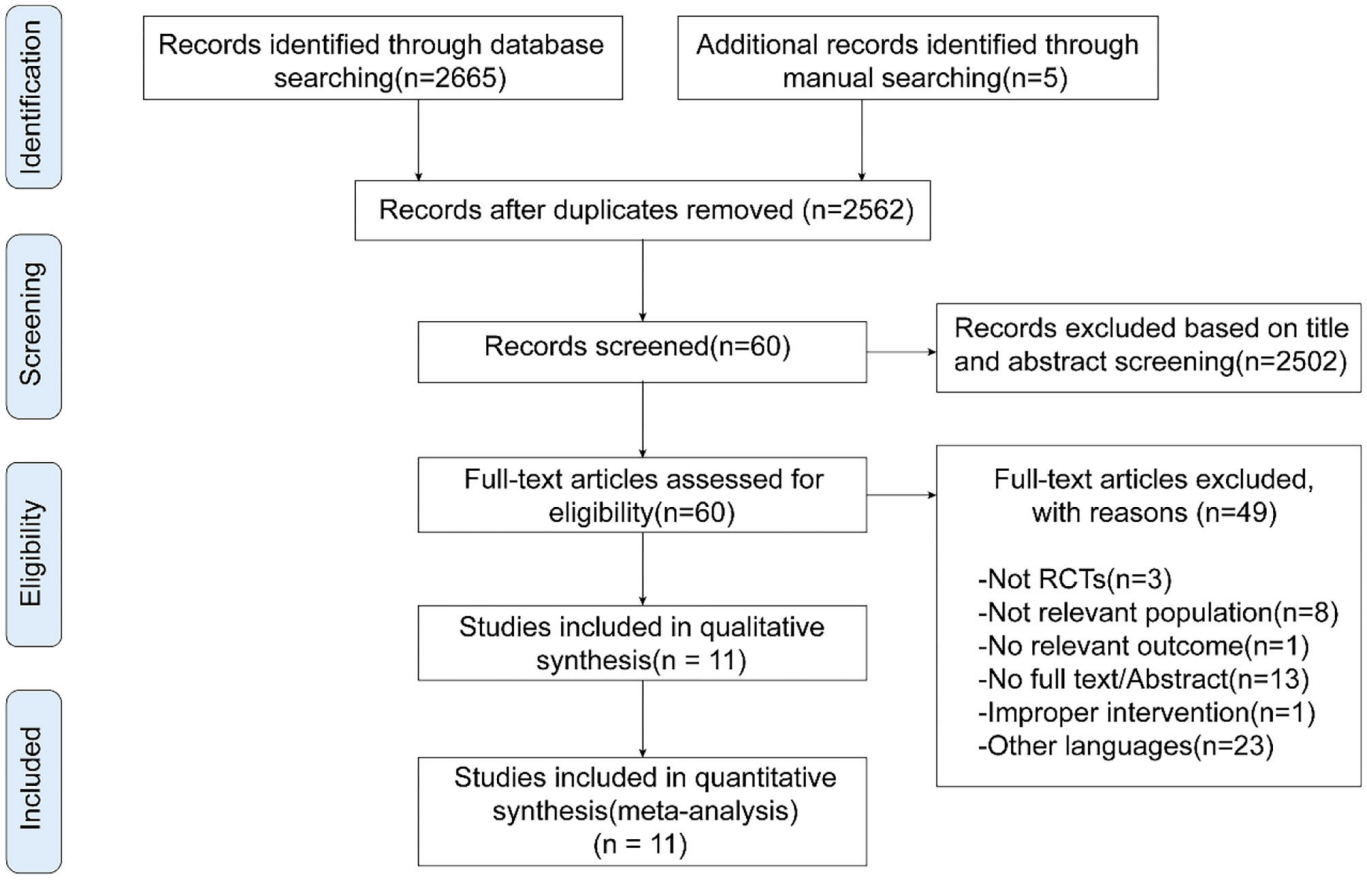
Flowchart of study selection.

A total of 2,670 potentially relevant records were acquired *via* electronic and hand search. We imported all the literature into EndNote X9 (Bld,12062) for de-duplication. Then we initially screened out 2,502 articles through title and abstract, and the remaining 60 were considered highly relevant. Finally, we further excluded 49 studies by means of reading full text according to our study selection criteria, leaving 11 studies (Cakmakçi, 2011; Gorji et al., 2014, 2015; Chaudhary, 2016; Savkin and Aslan, 2016; Khormizi and Azarniveh, 2017; Khajehlandi et al., 2018; Chen J. et al., 2020; Jung et al., 2020; Tyagi and Kumar, 2020; Wong et al., 2020) for inclusion in this analysis (Figure 1).

### Study Characteristics

All included studies were RCTs involving 393 adults with overweight or obesity. The studies were from Korea (Jung et al., 2020), Turkey (Cakmakçi, 2011; Savkin and Aslan, 2016), Iran (Gorji et al., 2014, 2015; Khormizi and Azarniveh, 2017; Khajehlandi et al., 2018), the USA (Wong et al., 2020), India (Chaudhary, 2016; Tyagi and Kumar, 2020), and China (Chen J. et al., 2020). These studies were published between 2011 and 2020, and the intervention duration varied from 8 to 24 weeks, with the sample size ranging from 20 to 61 participants. The participants were mostly female (77.1%). Nine studies (81.8%) only recruited women (Cakmakçi, 2011; Gorji et al., 2014, 2015; Savkin and Aslan, 2016; Khormizi and Azarniveh, 2017; Khajehlandi et al., 2018; Chen J. et al., 2020; Jung et al., 2020; Wong et al., 2020), and another two (18.2%) only recruited men (Chaudhary, 2016; Tyagi and Kumar, 2020). In terms of the definition of overweight and obesity, all 11 studies used BMI. Three studies recruited participants with hypertension (Wong et al., 2020), type 2 diabetes (Khormizi and Azarniveh, 2017), and depression (Chen J. et al., 2020), other studies recruited participants with no reported existing medical conditions. Regarding the measurement method of body composition, four studies (Gorji et al., 2014, 2015; Savkin and Aslan, 2016; Wong et al., 2020) used bioelectric impedance analysis device, three studies (Chaudhary, 2016; Khajehlandi et al., 2018; Tyagi and Kumar, 2020) used body composition analyzer, one study (Jung et al., 2020) used a dual-energy X-ray absorptiometry scanner, two studies (Cakmakçi, 2011; Chen J. et al., 2020) did not specify, and the remaining one (Khormizi and Azarniveh, 2017) did not measure. The characteristics of the included studies are summarized in Table 1.

**Table 1:** Characteristics of the included studies.

**References, country**	**Study design**	**Sample: population, sample size, mean age ± SD**	**Groups: sample size, mean age ± SD**	**Intervention**	**Intervention characteristics**	**Outcome measures**	**PEDro score**
Cakmakçi (2011), Turkey	RCT	Healthy sedentary obese women, *n* = 61	IG: *n* = 34, 36.15 ± 9.59 years	(a) IG: Pilates exercise	8 weeks, 4 sessions/week, 60 min/session	BW BMI BFP LBM WC	6
			CG: *n* = 27, 38.96 ± 10.02 years	(b) CG: without intervention			
							
Gorji et al. (2015), Iran	RCT	Sedentary overweight women aged between 37 and 45 years (BMI > 25 kg/m^2^), *n* = 60	IG: *n* = 15, 41.62 ± 2.05 years	(a) IG: Pilates exercise	8 weeks, 3 sessions/week, 60 min/session	BW BMI	6
			CG: *n* = 15, 40.62 ± 3.05 years	(b) CG: capsules containing starch powder, without other intervention			
			SG: *n* = 15, 40.62 ± 3.05 years	(c) SG: celery supplement, not included in meta-analysis			
			SPG: *n* = 15, 39.60 ± 2.70 years	(d) SPG: celery supplement plus Pilates exercise, not included in meta-analysis			
Savkin and Aslan (2016), Turkey	RCT	Sedentary overweight and obese women aged between 30 and 50 years (BMI ≥ 25 kg/m^2^), *n* = 37, (43.79 ± 4.88) years	IG: *n* = 19, 43.79 ± 4.88 years CG: *n* = 18, 39.67 ± 6.30 years	(a) IG: Pilates exercise (b) CG: normal lifestyle	8 weeks, 3 sessions/week, 90 min/session	BW BMI BFP LBM WC	6
Wong et al. (2020), USA	RCT	Obese young women with elevated blood pressure aged between 19 and 27 years and BMI (30–40 kg/m^2^), *n* = 28	IG: *n* = 14, 22.0 ± 3.74 years CG: *n* = 14, 23.0 ± 3.74 years	(a) IG: Pilates exercise (b) CG: normal lifestyle	12 weeks, 3 sessions/week, 60 min/session	BW BMI BFP LBM	8
Jung et al. (2020), Korea	RCT	Overweight and obese women aged between 34 and 60 years (BMI > 25 kg/m^2^), *n* = 32, (47.5 ± 7.5) years	IG: *n* = 10, 43.8 ± 8.6 years CG: *n* = 10, 51.6 ± 6.5 years	(a) IG: normoxic Pilates exercise (b) CG: normal lifestyle	12 weeks, 3 sessions/week, 50 min/session	BW BMI BFP	5
			HPG: *n* = 12, 47.2 ± 6.4 years	(c) HPG: hypoxic Pilates exercise, not included in meta-analysis			
Gorji et al. (2014), Iran	RCT	Sedentary overweight women aged between 35 and 45 years (BMI ≥ 25 kg/m^2^), *n* = 30	IG: *n* = 15, 39.60 ± 4.7 years CG: *n* = 15, 40.62 ± 3.05 years	(a) IG: Pilates exercise (b) CG: without intervention	8 weeks, 3 sessions/week, 60 min/session	BW BMI BFP	6
Khajehlandi et al. (2018), Iran	RCT	Overweight inactive women aged between 25 and 35 years, BMI (25–29 kg/m^2^), *n* = 28	IG: *n* = 14, 30.1 ± 4.0 years CG: *n* = 14, 29.6 ± 3.6 years	(a) IG: Pilates exercise (b) CG: normal lifestyle	12 weeks, 3 sessions/week, 60 min/session	BW BMI	6
Chaudhary (2016), India	RCT	Overweight male people aged between 30 and 45 years, *n* = 30	IG: *n* = 15 CG: *n* = 15	(a) IG: Pilates exercise (b) CG: without intervention	10 weeks, 5–6 sessions/week, 30–40 min/session	BFP	6
Tyagi and Kumar (2020), India	RCT	Obese male people aged between 20 and 45 years, *n* = 60	IG: *n* = 30 CG: *n* = 30	(a) IG: Pilates exercise (b) CG: without intervention	24 weeks	BW BMI BFP	6
Khormizi and Azarniveh (2017), Iran	RCT	Obese women with type 2 diabetes aged between 40 and 60 years (BMI > 30 kg/m^2^), *n* = 30, (51.9 ± 5.9) years	IG: *n* = 15, 51.06 ± 2.3 years CG: *n* = 15, 51.2 ± 3.7 years	(a) IG: Pilates exercise (b) CG: without intervention	8 weeks, 3 sessions/week, 60 min/session	BW BMI	7
Chen J. et al. (2020), China	RCT	Obese female college students (BMI ≥ 30 kg/m^2^), *n* = 39	IG: *n* = 20 CG: *n* = 19	(a) IG: Pilates exercise (b) CG: normal lifestyle	16 weeks, 3 sessions/week, 60 min/session	BW BMI BFP	5

*RCT, randomized controlled trials; SD, standard deviation; IG, intervention group; CG, control group; SG, supplement group; SPG, supplement plus Pilates group; HPG, hypoxic Pilates group; BW, body weight; BMI, body mass index; BFP, body fat percentage; LBM, lean body mass; WC, waist circumference*.

### Risk of Bias in Included Studies

The PEDro assessment indicated that the methodological quality of included studies was moderate (mean score = 6.09 ± 0.83; Table 2). All studies randomly allocated subjects, but only two studies (Savkin and Aslan, 2016; Wong et al., 2020) used concealed allocation. None of the studies employed subjects blinding and therapists blinding. However, only two studies (Khormizi and Azarniveh, 2017; Wong et al., 2020) used assessor blinding, which could lead to potential limitations. In addition, none of the studies had a loss to follow-up rate of more than 15%, and no study used intention-to-treat analysis.

**Table 2:** PEDro scores of the included studies.

**References**	**Eligibility criteria**	**Random allocation**	**Concealed allocation**	**Similar baseline**	**Blinding subjects**	**Blinding therapists**	**Blinding assessors**	**Dropout <15%**	**Intention to treat**	**Between-group statistics**	**Point measures**	**Total score**
Jung et al. (2020)	Yes	Yes	No	Yes	No	No	No	Yes	No	Yes	Yes	5
Cakmakçi (2011)	Yes	Yes	No	Yes	No	No	No	Yes	Yes	Yes	Yes	6
Gorji et al. (2015)	Yes	Yes	No	Yes	No	No	No	Yes	Yes	Yes	Yes	6
Savkin and Aslan (2016)	Yes	Yes	Yes	Yes	No	No	No	Yes	No	Yes	Yes	6
Wong et al. (2020)	Yes	Yes	Yes	Yes	No	No	Yes	Yes	Yes	Yes	Yes	8
Gorji et al. (2014)	Yes	Yes	No	Yes	No	No	No	Yes	Yes	Yes	Yes	6
Khajehlandi et al. (2018)	Yes	Yes	No	Yes	No	No	No	Yes	Yes	Yes	Yes	6
Chaudhary (2016)	Yes	Yes	No	Yes	No	No	No	Yes	Yes	Yes	Yes	6
Tyagi and Kumar (2020)	Yes	Yes	No	Yes	No	No	No	Yes	Yes	Yes	Yes	6
Khormizi and Azarniveh (2017)	Yes	Yes	No	Yes	No	No	Yes	Yes	Yes	Yes	Yes	7
Chen J. et al. (2020)	Yes	Yes	No	Yes	No	No	No	Yes	No	Yes	Yes	5

*PEDro, physiotherapy evidence database scale*.

### Analysis of Overall Effect Size

#### Primary Outcomes

##### Body Weight

**Figure 2:**
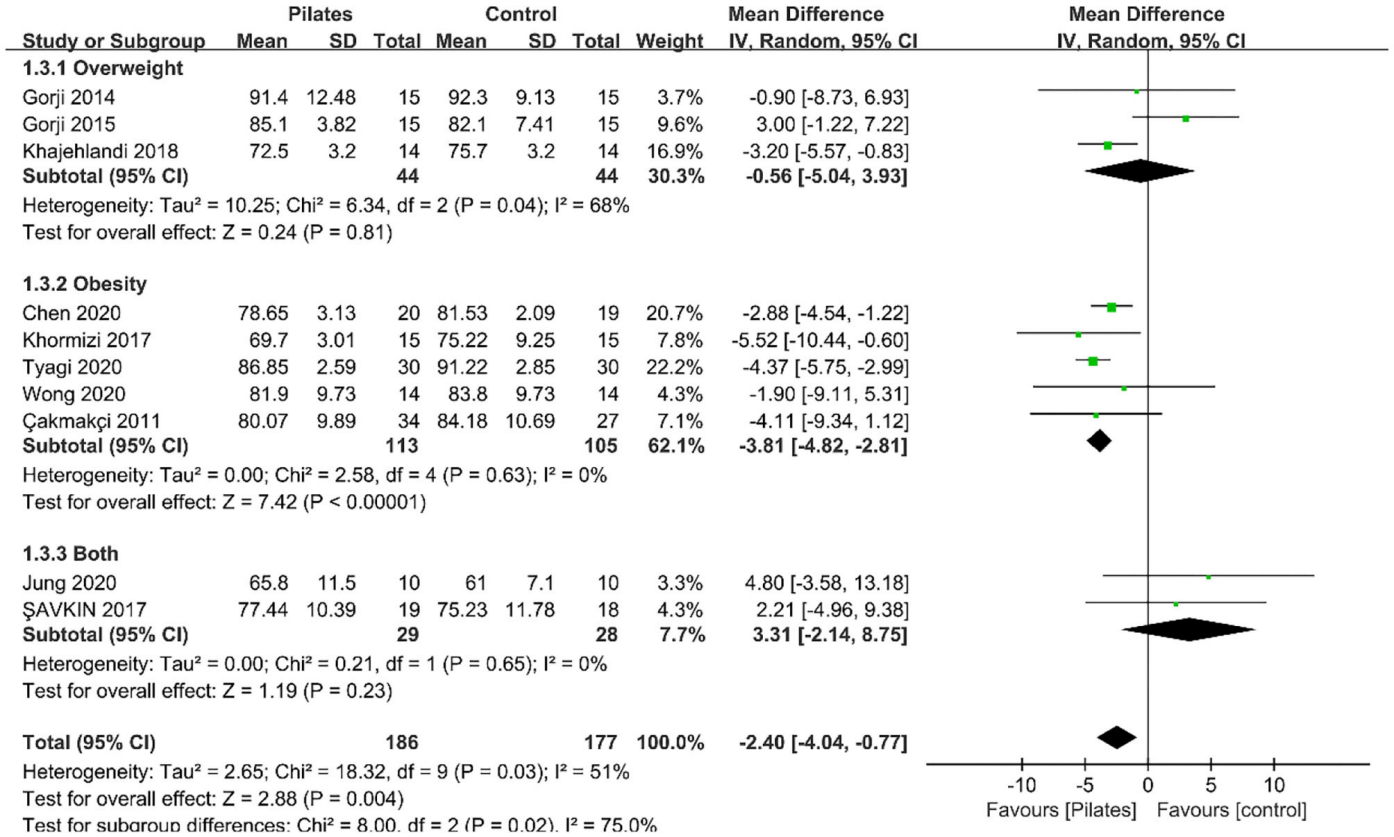
Forest plot of meta-analysis on body weight.

Ten studies recorded body weight outcomes. Meta-analysis results showed that Pilates significantly reduced body weight (MD = −2.40, 95% CI: [−4.04, −0.77], *P* = 0.004). Moderate heterogeneity was detected between studies (*P* = 0.03, *I*^2^ = 51%; Figure 2). Therefore, we conducted subgroup analyses for the results. Subgroup analysis based on intervention duration showed that a significant decrease was observed on body weight in studies with more than 10 weeks of duration (MD = −3.30, 95% CI: [−4.67, −1.92], *P* < 0.00001, *I*^2^ = 34%); whereas, no significant effect in studies with duration of 10 weeks or less (MD = −1.17, 95% CI: [−4.91, 2.58], *P* = 0.54, *I*^2^ = 55%). Moreover, the subgroup analysis according to participant type demonstrated that body weight dramatically decreased by Pilates in studies including participants with obesity only (MD = −3.81, 95% CI: [−4.82, −2.81], *P* < 0.00001, *I*^2^ = 0%), and no statistically significant effect in another two groups. The results of subgroup analyses are summarized in Table 3. Meta-regression analyses on body weight indicated that the intervention duration (β = −0.612, *P* = 0.189) and participant type (β = −0.16, *P* = 0.654) were not related to interstudy heterogeneity.

**Table 3:** Results of the subgroup analyses and overall analyses.

	**No. of datasets**	**No. of subjects**	**Meta-analysis MD (95% CI)**	***P*-value**	**Heterogeneity *Q* statistic**	***P*-value**	***I*^**2**^ (%)**	***P* between group**
**Body weight (kg)**								
Overall	10	363	−2.40 (−4.04, −0.77)	0.004	18.32	0.03	51	–
**Intervention duration**								
≤10 weeks	5	188	−1.17 (−4.91, 2.58)	0.54	8.82	0.07	55	0.29
>10 weeks	5	175	−3.30 (−4.67, −1.92)	<0.00001	6.08	0.19	34	
**Participant type**								
Overweight	3	88	−0.56 (−5.04, 3.93)	0.81	6.34	0.04	68	0.02
Obesity	5	218	−3.81 (−4.82, −2.81)	<0.00001	2.58	0.63	0	
Both	2	57	3.31 (−2.14, 8.75)	0.23	0.21	0.65	0	
**Body mass index (kg/m**^**2**^**)**								
Overall	10	363	−1.17 (−1.85, −0.50)	0.0006	23.04	0.006	61	–
**Intervention duration**								
≤10 weeks	5	188	−1.36 (−2.95, 0.24)	0.1	11.65	0.02	66	0.71
>10 weeks	5	175	−1.04 (−1.67, −0.40)	0.001	8.14	0.09	51	
**Participant type**								
Overweight	3	88	−0.83 (−1.59, −0.06)	0.03	0.79	0.67	0	0.44
Obesity	5	218	−1.35 (−2.33, −0.38)	0.007	18.84	0.0008	79	
Both	2	57	−0.03 (−1.93, 1.88)	0.98	0.01	0.93	0	
**Body fat percentage (%)**								
Overall	8	305	−4.22 (−6.44, −2.01)	0.002	59.72	<0.00001	88	–
**Intervention duration**								
≤10 weeks	4	158	−6.39 (−10.65, −2.12)	0.003	40.47	<0.00001	93	0.10
>10 weeks	4	147	−2.77 (−3.65, −1.88)	<0.00001	2.35	0.50	0	
**Participant type**								
Overweight	2	60	−9.82 (−20.16, 0.53)	0.06	28.24	<0.00001	96	0.04
Obesity	4	188	−3.56 (−5.07, −2.04)	<0.00001	9.02	0.03	67	
Both	2	57	−0.12 (−2.82, 2.58)	0.93	0.13	0.72	0	

*Both, overweight and obesity; MD, mean difference*.

##### Body Mass Index

**Figure 3:**
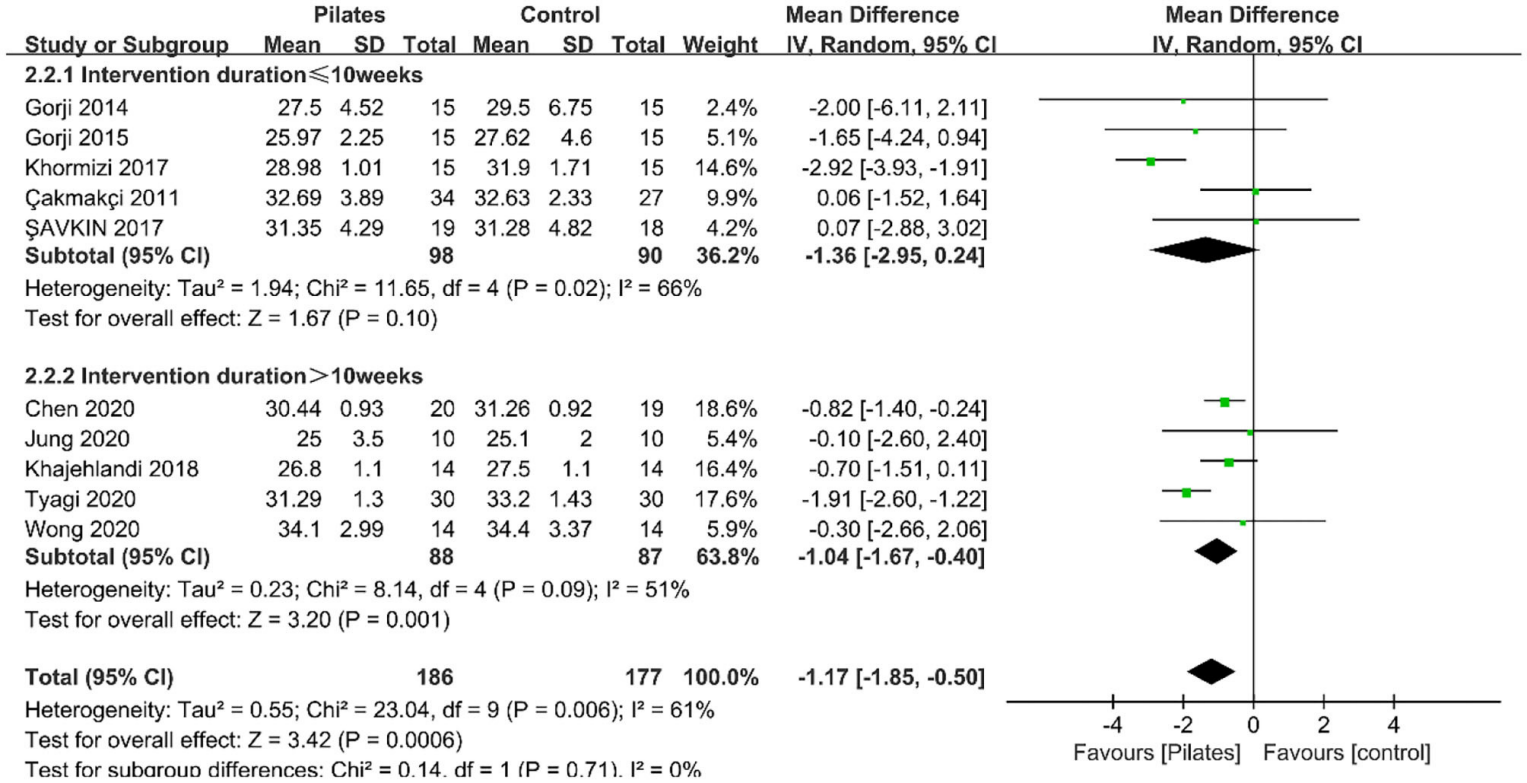
Forest plot of meta-analysis on BMI.

Ten studies recorded outcomes for BMI. Meta-analysis results indicated that Pilates remarkably decreased BMI (MD = −1.17, 95% CI: [−1.85, −0.50], *P* = 0.0006) and a moderate heterogeneity was detected between studies (*P* = 0.006, *I*^2^ = 61%; Figure 3). Subgroup analysis based on intervention duration revealed that a significant reduction was observed on BMI in studies with more than 10 weeks of duration (MD = −1.04, 95% Cl: [−1.67, −0.40], *P* = 0.001, *I*^2^ = 51%); however, no significant effect in studies with duration of 10 weeks or less (MD = −1.36, 95% Cl: [−2.95, 0.24], *P* = 0.10, *I*^2^ = 66%). Additionally, the subgroup analysis according to participant type demonstrated that a remarkable decrease on BMI in studies including participants with overweight only or ones with obesity only (Table 3), which may be the reason for the excessive individual differences. Meta-regression analyses on BMI showed that the intervention duration (β = −0.142, *P* = 0.761) and participant type (β = −0.17, *P* = 0.613) were not correlated to interstudy heterogeneity.

##### Body Fat Percentage

**Figure 4:**
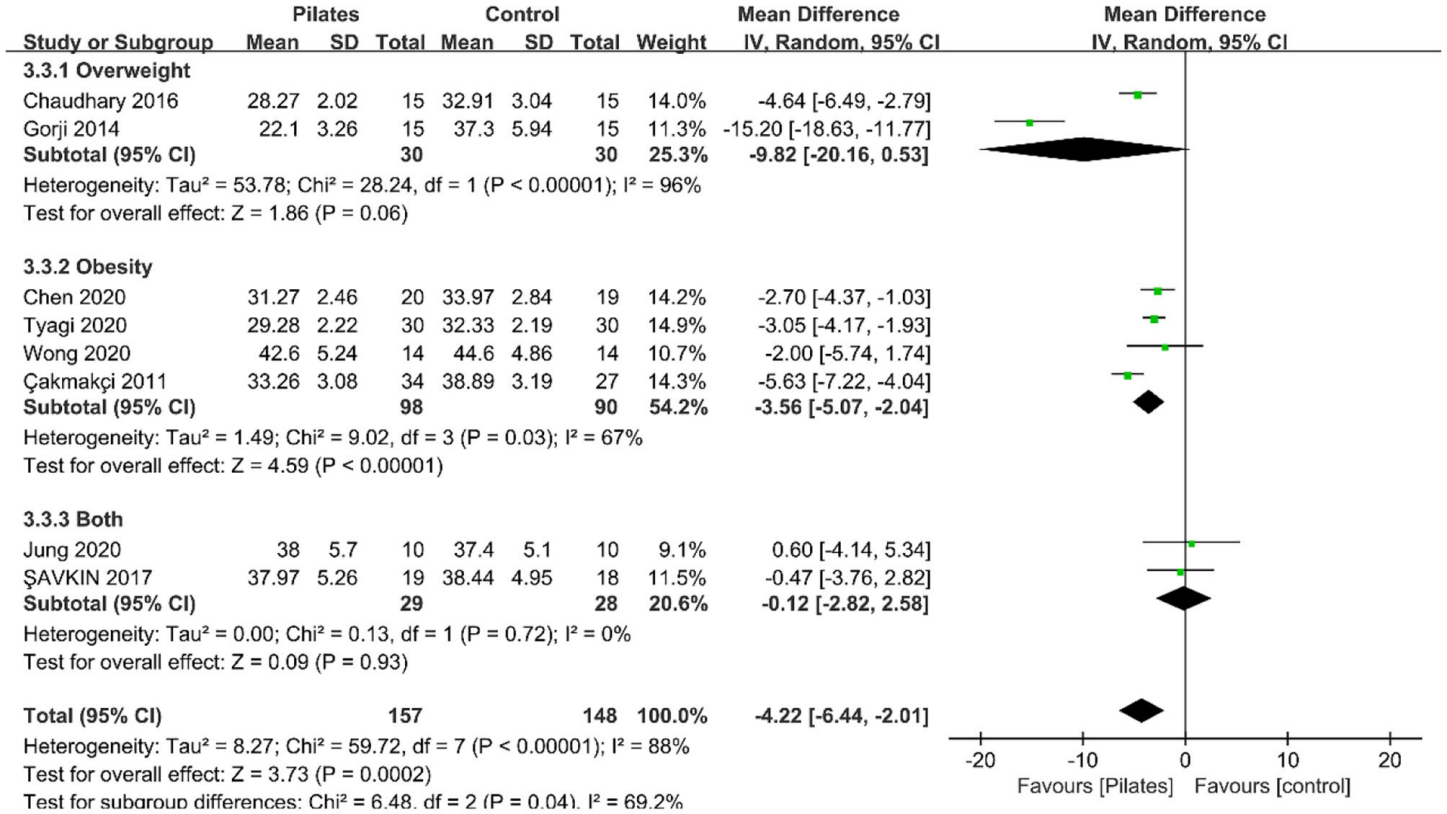
Forest plot of meta-analysis on body fat percentage.

Eight studies recorded outcomes for body fat percentage (BFP). Meta-analysis results revealed that Pilates significantly reduced BFP (MD = −4.22, 95% CI: [−6.44, −2.01], *P* = 0.0002), but a high heterogeneity was detected between studies (*P* < 0.00001, *I*^2^ = 88%; Figure 4). The subgroup analysis according to participant type revealed that a significant reduction on BFP in studies including participants with obesity only (MD = −3.56, 95% CI: [−5.07, −2.04], *P* < 0.00001, *I*^2^ = 67%), and no statistically significant effect in another two groups (Table 3).

##### Lean Body Mass and Waist Circumference

**Figure 5:**
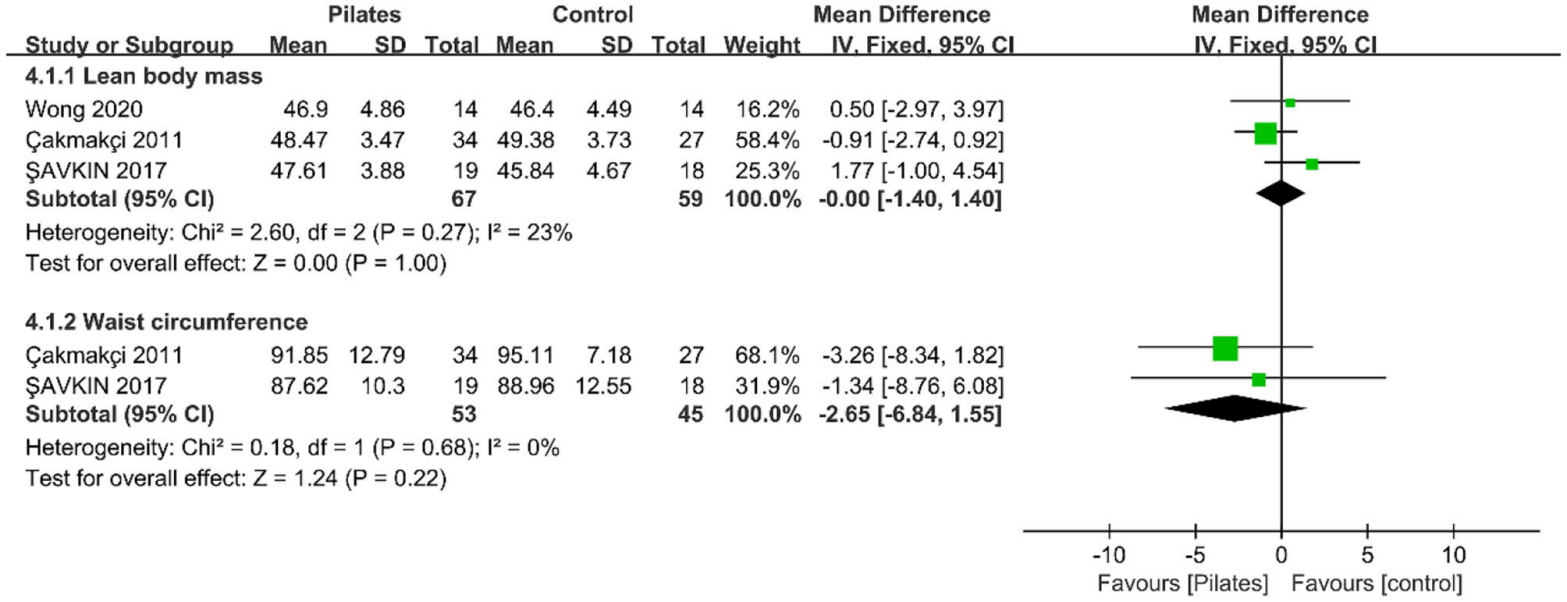
Forest plot of meta-analysis on lean body mass and waist circumference.

The analysis of three studies indicated that no significant effect on lean body mass (LBM) after Pilates (MD = −0.00, 95% CI: [−1.40, 1.40], *P* = 1.00), and a very low heterogeneity was seen between studies (*P* = 0.27, *I*^2^ = 23%). In addition, the meta-analysis of two studies demonstrated that waist circumference (WC) did not decrease after Pilates (MD = −2.65, 95%CI: [−6.84, 1.55], *P* = 0.22, *I*^2^ = 0%; Figure 5).

##### Secondary Outcomes

We considered safety as secondary outcomes in this study; however, no adverse events following Pilates intervention were reported in the included studies.

### Sensitivity Analysis

The leave-one-out approach was used for sensitivity analysis of each outcomes (Patsopoulos et al., 2008; Supplementary Table 2). The analysis results indicated that the meta-analysis results for BMI and BFM did not alter when each study was removed in turn, and that the findings were robust. In the meta-analysis of BMI outcome, the removal of studies conducted by Khormizi and Azarniveh (2017) significantly reduced the heterogeneity, suggesting that this study could be the potential source of heterogeneity. Additionally, the meta-analysis results for body weight changed from significant (*P* < 0.05) to non-significant (*P* = 0.06) when the study by Chen J. et al. (2020) was removed, suggesting that this study was primarily responsible for the between-study heterogeneity.

### Publication Bias

**Figure 6:**
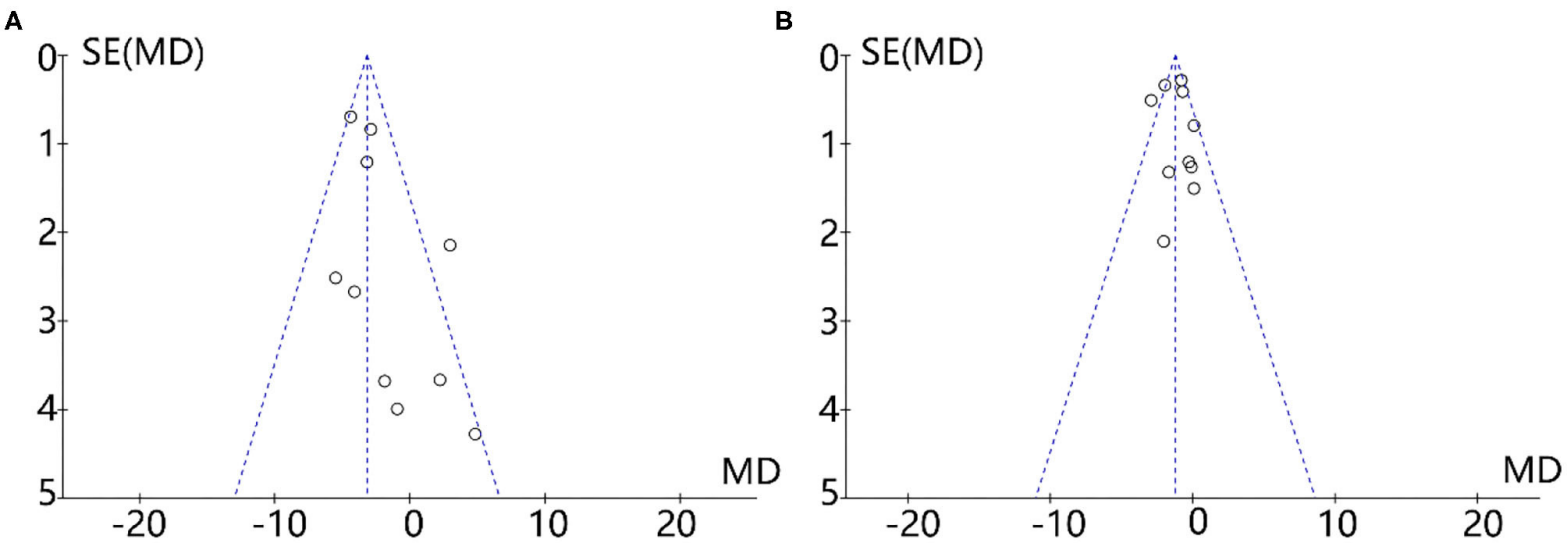
Funnel plots of 10 RCTs recording body weight **(A)** and BMI **(B)** outcomes.

Visual inspection of funnel plots revealed no asymmetry (Figure 6), and the results from Egger's and Begg's test indicated that no evidence for publication bias was detected for body weight (Begg's test, *P* = 0.59; Egger's test, *P* = 0.35) and BMI (Begg's test, *P* = 0.37; Egger's test, *P* = 0.55).

### Quality of Evidence

Regarding the assessment of the quality of evidence, there is low to moderate evidence in body weight and BMI, very low to low evidence in BFP, low evidence in lean body mass, and moderate evidence in waist circumference. This indicates that more researches may have a significant impact on the results of the effect estimate and even may change the results. The detailed information was presented in Supplementary Table 3.

## Discussion

### Summary of Main Results

We performed this study to evaluate scientific evidence for the efficacy of Pilates in improving body weight and body composition in adults with overweight or obesity. The analyses combined 11 studies involving a total of 393 adults with overweight or obesity (90 men and 303 women). Outcome measures were body weight, BMI, BFP, lean body mass, and WC. Overall results revealed that Pilates leads to a significant decrease in body weight, BMI, and BFP in adults with overweight or obesity. The reduction in body weight and BFP appears to be more pronounced in studies including participants with obesity only, and the efficacy of Pilates for the improvement of body weight and BMI appears to be more evident in longer intervention duration. These findings could have significant implications for the promotion of exercise interventions for overweight and obesity management. We also found that Pilates has no remarkable effect on WC and lean body mass. However, given the low to moderate overall quality of the evidence, we are still unable to draw a definite conclusion. Furthermore, since no trials reported adverse events, we could not judge the safety of Pilates.

### Comparison With Other Reviews

To our knowledge, this is the first systematic review and meta-analysis to investigate the effects of Pilates on body weight and body composition in adults with overweight or obesity. In the previous reviews, few studies systematically analyzed and assessed the effects of Pilates on body weight and body composition. Aladro-Gonzalvo et al. (2012) conducted a systematic review to determine the effects of Pilates on body composition in several populations; the results suggested that there is insufficient evidence indicating a positive effect of Pilates on body weight and body composition. This finding is inconsistent with our review, and the main reason that might explain this is the poor methodological quality of the included studies. In another systematic review (Kamioka et al., 2016), there was some evidence that Pilates could reduce body fat and increase fat free mass. Although this is partially consistent with our findings, the authors of this review did not perform a meta-analysis, which limited the comparison with our review. The most recent review (de Souza Cavina et al., 2020) showed no evidence for the efficacy of Pilates in improving body weight and body composition in general population including overweight and obese individuals; the results conflicted with our present review, and insufficient training intensity and not performing diet control could be the main reasons for these results.

### How the Intervention Might Work

Overweight and obesity, which are serious public health problems worldwide, are associated with many comorbidities such as insulin resistance and diabetes (Diabetes Prevention Program Research Group, 2002; Look AHEAD Research Group, 2003), hypertension and cardiovascular disease (Ozbey et al., 2002), metabolic syndrome (Frank et al., 2005), depression, and also cancer (Field et al., 2001). While overweight and obesity are being prevented and treated worldwide, they continue to be highly prevalent conditions that impose a substantial economic burden (Kushner and Kahan, 2018). Therefore, growing attention has been poured to the problems of body weight and a mounting number of medical staff have attached importance to develop available and affordable therapies for reducing body weight. Some studies indicated that exercise could improve the functions of several body systems, thereby improving body weight, body composition, cardiometabolic risk factors, and emotional health status (Jakicic et al., 2018; Garber, 2019), so exercise is widely used to reduce body weight in clinical practice. As for Pilates, it is often considered an alternative form of exercise. It was reported that completion of 30–45 min Pilates exercise could produce sufficient stimuli to induce positive changes in energy expenditure, thus reducing body weight (Olson et al., 2004). Moreover, since Pilates exercise involves employing different types of resistance (Lange et al., 2000), it could enhance the muscle strength of upper body parts, lower body parts, and abdomen (Bergamin et al., 2015) and could thus contribute to improving body composition. Besides exercise, Pilates also involves relaxation, concentration, and breath control. Thus, Pilates has been shown to effectively improve depression and anxiety (Vancini et al., 2017; Fleming and Herring, 2018; Aibar-Almazán et al., 2019), which might in turn improve overweight or obesity caused by emotional factors (Jantaratnotai et al., 2017).

### Limitations

However, this present review had several potential limitations. Firstly, the overall effect sizes could have been affected by lack of concealed allocation (9 out of 11 studies), inability to use therapists/subjects blinding (all studies) or assessors blinding (9 out of 11 studies). Secondly, publication bias might have been a factor, as this review retrieved only relevant articles in the English and Chinese language within a limited number of electronic databases. Thirdly, given the small size of overall samples, some conclusions should be considered preliminary and even might be biased. Fourthly, there was a moderate to high heterogeneity between studies, while several subgroup analyses could explain the heterogeneity to some extent; however, considerable heterogeneity remained unclear. This may be partially because of the differences in participants (e.g., age, gender, race, lifestyle), Pilates intervention (e.g., exercise modalities, duration, dose, intensity), and the measuring method of outcomes. In addition, it remained unclear whether Pilates was more effective than other exercises for improving body weight and body composition as the included studies did not involve other exercise interventions.

### Implications for Clinical Practice

This present meta-analysis showed that Pilates could improve body weight, BMI, and BFP in adults with overweight or obesity. Although the methodological quality of the included RCTs is poor, Pilates could still be preliminarily considered an effective intervention to treat overweight and obesity. It has been shown that people with overweight or obesity find it difficult to adhere to physical activities targeting weight loss (Burgess et al., 2017), mainly due to the monotony of physical activities (Vancini et al., 2017); these people are however more likely to practice Pilates (de Souza Cavina et al., 2020). Therefore, Pilates could be specifically considered an alternative to other forms of physical exercise for overweight or obese people who do not abide by recommended physical exercise regimens.

### Implications for Further Research

Given the poor methodological quality of the majority of RCTs and the limited strength of evidence, more rigorous and robust methods are recommended to use in future studies. Firstly, the reporting of future Pilates RCTs should be improved and strictly follow standard reporting guidelines such as CONSORT (Schulz et al., 2010). Secondly, future RCTs should further ensure the use of concealed allocation, therapists/subjects blinding, intention-to-treat analysis, and assessors blinding. Thirdly, to reduce potential publication bias, the researchers are recommended to register beforehand in the clinical trial registry before recruiting participants. Fourthly, there were no adverse events described in any of the included studies, considering that safety is critical to the evaluation of therapies, future RCTs should improve the reporting of safety. Fifthly, future RCTs should compare Pilates with other interventions (e.g., aerobic exercise, resistance exercise) to evaluate the potential benefits of Pilates for overweight and obesity. In addition, there is evidence that diet and exercise play an important role in weight loss and promoting positive changes in body composition (Clark, 2015). Considering that diet is an uncontrollable factor, in order to eliminate the significant interference of diet on the results, we excluded studies with dietary intervention in this study to determine the effects of Pilates on body weight and body composition in adults with overweight or obesity. Some studies demonstrated that dietary intervention plus exercise resulted in an even greater reduction in body weight and body composition profile parameters than dietary interventions alone (Figueroa et al., 2013; Cheng et al., 2018). In view of this, it remained uncertain whether Pilates plus diet intervention was more effective than Pilates interventions alone in improving body weight and body composition, and further researches are needed to confirm it.

## Conclusion

In conclusion, this present study indicated that Pilates lead to a remarkable decrease in body weight, BMI, and BFP in adults with overweight or obesity. No significant effect on WC and LBM. Large-scale and well-designed RCTs with improved methodology and reporting are recommended to further elucidate the effectiveness of Pilates for treating overweight and obesity.

## Data Availability Statement

The original contributions presented in the study are included in the article/Supplementary Material, further inquiries can be directed to the corresponding author/s.

## Author Contributions

YW and XX conceived and designed the study. YW and ZC performed the literature search, data extraction, and data analysis. ZW and XY helped with the data analysis and reviewed the manuscript for important content. XX supervised the study. All authors have read and approved the final version of the submitted version.

## Conflict of Interest

The authors declare that the research was conducted in the absence of any commercial or financial relationships that could be construed as a potential conflict of interest.
