# Supplementary material for: Pilates for Overweight or Obesity: A Meta-Analysis
Source: Front Physiol. 2021 Mar 11;12:643455. doi: 10.3389/fphys.2021.643455 (PMC7992419; doi:10.3389/fphys.2021.643455)
Supplement: Supplementary file 1 [file Data_Sheet_1.PDF]

Supplementary Material

Table S1-Search strategy used

**PubMed**	#1 ((((((((((("Overweight"[Mesh]) OR ("Obesity"[Mesh])) OR (Overweight [Title/Abstract])) OR(Obesity[Title/Abstract])) OR (adipose tissue hyperplasia[Title/ Abstract])) OR(adipositas[Title/Abstract])) OR (adiposity[Title/Abstract])) OR (alimentary obesity[Title/Abstract])) OR (body weight, excess[Title/Abstract])) OR (corpulency[Title/Abstract])) OR (fat overload syndrome[Title/Abstract])) OR (nutritional obesity[Title/Abstract])) OR (obesitas[Title/Abstract])	363,778
	#2 (((((((("Exercise Movement Techniques"[Mesh]) OR (Movement Techniques, Exercise[Title/Abstract])) OR (Exercise Movement Technics[Title/Abstract])) OR (Pilates-Based Exercises[Title/Abstract])) OR (Exercises, Pilates-Based[Title/ Abstract])) OR (Pilates Based Exercises[Title/Abstract])) OR (Pilates Training[Title/ Abstract])) OR (Training, Pilates[Title/Abstract])) OR (Pilates[Title/Abstract])	54,542
	#3 (randomized controlled trial[pt] OR controlled clinical trial[pt] OR randomized[tiab] OR placebo[tiab] OR drug therapy[sh] OR randomly[tiab] OR trial[tiab] OR groups[tiab] NOT (animals [mh] NOT humans [mh]))	4,265,196
	#4 #1 AND #2 AND #3	2,432
**Embase**	#1 'obesity'/exp	539,419
	#2 'overweight':ab,ti OR 'adipose tissue hyperplasia':ab,ti OR adipositas:ab,ti OR adiposity:ab,ti OR 'alimentary obesity':ab,ti OR 'body weight, excess':ab,ti OR corpulency:ab,ti OR 'fat overload syndrome':ab,ti OR 'nutritional obesity':ab,ti OR obesitas:ab,ti OR obesity:ab,ti	424,628
	#3 #1 OR #2	610,935
	#4 'pilates'/exp	725
	#5 'pilates exercise':ab,ti OR 'movement techniques, exercise':ab,ti OR 'exercise movement technics':ab,ti OR 'pilates-based exercises':ab,ti OR 'exercises, pilates-based':ab,ti OR 'pilates based exercises':ab,ti OR 'pilates training':ab,ti OR 'training, pilates':ab,ti OR 'exercise movement techniques':ab,ti OR pilates:ab,ti	819
	#6 #4 OR #5	950
	#7 'randomized controlled trial'/exp OR 'controlled clinical trial'/exp OR 'randomized':ti,ab OR 'placebo':ti,ab OR 'drug therapy':lnk OR 'randomly':ti,ab OR 'trial':ti,ab OR 'groups':ti,ab	7,596,029
	#8 #3 AND #6 AND #7	33
**Cochrane Library**	#1 MeSH descriptor: [Overweight] explode all trees	16,383
	#2 MeSH descriptor: [Obesity] explode all trees	13,803
	#3 (adipose tissue hyperplasia):ti,ab,kw OR (adipositas):ti,ab,kw OR (adiposity): ti,ab, kw OR (alimentary obesity):ti,ab,kw OR (body weight, excess):ti,ab,kw OR (corpulency):ti,ab,kw OR (fat overload syndrome):ti,ab,kw OR (nutritional obesity): ti,ab,kw OR (obesitas):ti,ab,kw OR (obesity):ti,ab,kw OR (overweight):ti,ab,kw (Word variations have been searched)	49,878
	#4 #1 OR #2 OR #3 OR	49,952
	#5 MeSH descriptor: [Exercise Movement Techniques] explode all trees	2,108
	#6 (Movement Techniques, Exercise):ti,ab,kw OR (Exercise Movement Technics): ti,ab,kw OR (Pilates-Based Exercises):ti,ab,kw OR (Pilates Based Exercises):ti,ab,kw OR (Training, Pilates):ti,ab,kw OR (Pilates Training):ti,ab,kw OR(Exercises, Pilates -Based):ti,ab,kw OR (Pilates):ti,ab,kw (Word variations have been searched)	1,532
	#7 #5 OR #6	3,391
	#8 #4 AND #7	127
**Web of Science**	#1 TS=(overweight OR obesity OR adipose tissue hyperplasia OR adipositas OR adiposity OR alimentary obesity OR body weight, excess OR corpulency OR fat overload syndrome OR nutritional obesity OR obesitas)	353,327
	#2 TS=(Exercise Movement Techniques OR Movement Techniques, Exercise OR Exercise Movement Technics OR Pilates-Based Exercises OR Exercises, Pilates-Based OR Pilates Based Exercises OR Pilates Training OR Training, Pilates OR	

	Pilates)<br>#3 TS=(randomized controlled trial OR randomized)<br>#4 #1 AND #2 AND #3	1,587<br>787,985<br>12
**CNKI**	（主题：肥胖）OR（主题：超重）OR（篇关摘：肥胖）OR（篇关摘：超重）AND（（主题：普拉提) OR（主题：Pilates）OR（篇关摘：普拉提）OR（篇关摘：Pilates））	61

Table S2-Results of the sensitivity analyses

1. Sensitivity analysis for body weight

References	MD (95%CI)	*P*	I^2
Chen2020	-2.03 (-4.17, 0.11)	0.06	56%
Gorji2014	-2.43 (-4.14, -0.72)	0.005	56%
Gorji2015	-3.31 (-4.47, -2.15)	< 0.00001	17%
Jung2020	-2.73 (-4.26, -1.20)	< 0.00001	46%
Khajehlandi2018	-2.09 (-4.07, -0.10)	0.04	56%
Khormizi2017	-2.11 (-3.84, -0.37)	0.02	54%
Tyagi2020	-2.37 (-3.53, -1.21)	< 0.0001	41%
Wong2020	-2.38 (-4.11, -0.65)	0.007	56%
Çakmakçi2011	-2.21 (-3.98, -0.44)	0.01	56%
ŞAVKIN2017	-2.65 (-4.26, -1.03)	0.001	50%

2. Sensitivity analysis for body mass index（BMI）

References	MD (95%CI)	*P*	I^2
Chen2020	-1.22 (-2.03, -0.41)	0.003	60%
Gorji2014	-1.15 (-1.84, -0.45)	0.001	65%
Gorji2015	-1.14 (-1.85, -0.43)	0.002	65%
Jung2020	-1.23 (-1.93, -0.53)	0.0006	64%
Khajehlandi2018	-1.25 (-2.03, -0.47)	0.002	62%
Khormizi2017	-1.03 (-1.40, -0.67)	< 0.00001	28%
Tyagi2020	-1.01 (-1.77, -0.26)	0.0009	57%
Wong2020	-1.23 (-1.93, -0.52)	0.0007	64%
Çakmakçi2011	-1.31 (-2.01, -0.62)	0.0002	61%
ŞAVKIN2017	-1.23 (-1.92, -0.53)	0.0005	64%

3. Sensitivity analysis for body fat percentage

References	MD (95%CI)	*P*	I^2
Chaudhary2016	-4.15 (-6.77, -1.53)	0.002	90%
Chen2020	-4.46 (-7.09, -1.82)	0.0009	90%
Gorji2014	-3.18 (-4.50, -1.86)	< 0.00001	64%
Jung2020	-4.70 (-7.01, -2.39)	< 0.0001	89%
Tyagi2020	-4.40 (-7.24, -1.56)	0.002	89%
Wong2020	-4.49 (-6.89, -2.09)	0.0002	90%
Çakmakçi2011	-3.98 (-6.59, -1.38)	0.003	89%
ŞAVKIN2017	-4.71 (-7.07, -2.36)	< 0.0001	89%

Table S3- Evidence quality assessment according to GRADE.

Pilates for overweight and obesity

Patient or population: patients with overweight or obesity
Settings:
Intervention: Pilates

Outcomes	Illustrative comparative risks* (95% CI)		Relative effect (95% CI)	No of Participants (studies)	Quality of the evidence (GRADE)	Comments
	Assumed risk	Corresponding risk				
	**Control**	**Pilates**				
**Body weight (Intervention duration≤10weeks)**		The mean body weight (intervention duration≤10weeks) in the intervention groups was **1.17 lower** (4.91 lower to 2.58 higher)		188 (5 studies)	⊕⊕⊝⊝ **low**[1,2]	This is a small effect that may be clinically relevant in this patient group
**Body weight (Intervention duration＞10weeks)**		The mean body weight (intervention duration＞10weeks) in the intervention groups was **3.3 lower** (4.67 to 1.92 lower)		175 (5 studies)	⊕⊕⊕⊝ **moderate**[2]	This is a moderate effect that is clinically relevant in this patient group
**Body weight (participant type: overweight)**		The mean body weight (participant type: overweight) in the intervention groups was **0.56 lower** (5.04 lower to 3.93 higher)		88 (3 studies)	⊕⊕⊕⊝ **moderate**[2]	This is a moderate effect that is clinically relevant in this patient group
**Body weight (participant type: obesity)**		The mean body weight (participant type: obesity) in the intervention groups was **3.81 lower** (4.82 to 2.81 lower)		218 (5 studies)	⊕⊕⊕⊝ **moderate**[2]	This is a moderate effect that is clinically relevant in this patient group
**Body weight (participant type: overweight and obesity)**		The mean body weight (participant type: overweight and obesity) in the intervention groups was **3.31 higher** (2.14 lower to 8.75 higher)		57 (2 studies)	⊕⊕⊕⊝ **moderate**[2]	This is a moderate effect that is clinically relevant in this patient group
**Body mass index (Intervention duration≤10weeks)**		The mean body mass index (intervention duration≤10weeks) in the intervention groups was **1.36 lower** (2.95 lower to 0.24 higher)		188 (5 studies)	⊕⊕⊝⊝ **low**[1,2]	This is a small effect that may be clinically relevant in this patient group
**Body mass index (Intervention duration＞10weeks)**		The mean body mass index (intervention duration＞10weeks) in the intervention groups was **1.04 lower** (1.67 to 0.4 lower)		175 (5 studies)	⊕⊕⊝⊝ **low**[1,2]	This is a small effect that may be clinically relevant in this patient group
**Body mass index (participant type: overweight)**		The mean body mass index (participant type: overweight) in the intervention groups was **0.83 lower** (1.59 to 0.06 lower)		88 (3 studies)	⊕⊕⊕⊝ **moderate**[2]	This is a moderate effect that is clinically relevant in this patient group
**Body mass index (participant type: obesity)**		The mean body mass index (participant type: obesity) in the intervention groups was **1.35 lower** (2.33 to 0.38 lower)		218 (5 studies)	⊕⊕⊝⊝ **low**[1,2]	This is a small effect that may be clinically relevant in this patient group

**Body mass index (participant type: overweight and obesity)**		The mean body mass index (participant type: overweight and obesity) in the intervention groups was **0.03 lower** (1.93 lower to 1.88 higher)		57 (2 studies)	⊕⊕⊕⊝ **moderate**[2]	This is a moderate effect that is clinically relevant in this patient group
**Body fat percentage (Intervention duration≤10weeks)**		The mean body fat percentage (intervention duration≤10weeks) in the intervention groups was **6.39 lower** (10.65 to 2.12 lower)		158 (4 studies)	⊕⊝⊝⊝ **very low**[1,2,3]	This is a weak effect that is clinically relevant in this patient group
**Body fat percentage (Intervention duration＞10weeks)**		The mean body fat percentage (intervention duration＞10weeks) in the intervention groups was **2.77 lower** (3.65 to 1.88 lower)		147 (4 studies)	⊕⊕⊝⊝ **low**[2,3]	This is a small effect that may be clinically relevant in this patient group
**Body fat percentage (participant type: overweight)**		The mean body fat percentage (participant type: overweight) in the intervention groups was **9.82 lower** (20.16 lower to 0.53 higher)		60 (2 studies)	⊕⊝⊝⊝ **very low**[1,2,3]	This is a weak effect that is clinically relevant in this patient group
**Body fat percentage (participant type: obesity)**		The mean body fat percentage (participant type: obesity) in the intervention groups was **3.56 lower** (5.07 to 2.04 lower)		188 (4 studies)	⊕⊝⊝⊝ **very low**[1,2,3]	This is a weak effect that is clinically relevant in this patient group
**Body fat percentage (participant type: overweight and obesity)**		The mean body fat percentage (participant type: overweight and obesity) in the intervention groups was **0.12 lower** (2.82 lower to 2.58 higher)		57 (2 studies)	⊕⊕⊝⊝ **low**[2,3]	This is a small effect that may be clinically relevant in this patient group
**Lean body mass**		The mean lean body mass in the intervention groups was **0.13 higher** (1.53 lower to 1.8 higher)		126 (3 studies)	⊕⊕⊝⊝ **low**[2,3]	The difference is not statistically or clinically significant
**Waist circumference**		The mean waist circumference in the intervention groups was **2.65 lower** (6.84 lower to 1.55 higher)		98 (2 studies)	⊕⊕⊕⊝ **moderate**[2]	The difference is not statistically or clinically significant
**Adverse events**	See comment	See comment	Not estimable	0 (0)	See comment	None of the included studies reported adverse events

*The basis for the **assumed risk** (e.g. the median control group risk across studies) is provided in footnotes. The **corresponding risk** (and its 95% confidence interval) is based on the assumed risk in the comparison group and the **relative effect** of the intervention (and its 95% CI).
CI: Confidence interval; **OR:** Odds ratio;

GRADE Working Group grades of evidence
High quality: Further research is very unlikely to change our confidence in the estimate of effect.
Moderate quality: Further research is likely to have an important impact on our confidence in the estimate of effect and may change the estimate.
Low quality: Further research is very likely to have an important impact on our confidence in the estimate of effect and is likely to change the estimate.
Very low quality: We are very uncertain about the estimate.

[1] Downgraded one level due to inconsistency ($I^2 > 50\%$).

[2] Downgraded one level due to imprecision (fewer than 400 participants, total).

[3] Downgraded one level due to different measurement method.

PRISMA 2009 Checklist

Section/topic	#	Checklist item	Reported on page #
**TITLE**			
Title	1	Identify the report as a systematic review, meta-analysis, or both.	1
**ABSTRACT**			
Structured summary	2	Provide a structured summary including, as applicable: background; objectives; data sources; study eligibility criteria, participants, and interventions; study appraisal and synthesis methods; results; limitations; conclusions and implications of key findings; systematic review registration number.	1
**INTRODUCTION**			
Rationale	3	Describe the rationale for the review in the context of what is already known.	2
Objectives	4	Provide an explicit statement of questions being addressed with reference to participants, interventions, comparisons, outcomes, and study design (PICOS).	2
**METHODS**			
Protocol and registration	5	Indicate if a review protocol exists, if and where it can be accessed (e.g., Web address), and, if available, provide registration information including registration number.	2
Eligibility criteria	6	Specify study characteristics (e.g., PICOS, length of follow-up) and report characteristics (e.g., years considered, language, publication status) used as criteria for eligibility, giving rationale.	3
Information sources	7	Describe all information sources (e.g., databases with dates of coverage, contact with study authors to identify additional studies) in the search and date last searched.	2
Search	8	Present full electronic search strategy for at least one database, including any limits used, such that it could be repeated.	3
Study selection	9	State the process for selecting studies (i.e., screening, eligibility, included in systematic review, and, if applicable, included in the meta-analysis).	3
Data collection process	10	Describe method of data extraction from reports (e.g., piloted forms, independently, in duplicate) and any processes for obtaining and confirming data from investigators.	4

Data items	11	List and define all variables for which data were sought (e.g., PICOS, funding sources) and any assumptions and simplifications made.	3
Risk of bias in individual studies	12	Describe methods used for assessing risk of bias of individual studies (including specification of whether this was done at the study or outcome level), and how this information is to be used in any data synthesis.	4
Summary measures	13	State the principal summary measures (e.g., risk ratio, difference in means).	4
Synthesis of results	14	Describe the methods of handling data and combining results of studies, if done, including measures of consistency (e.g., I^2) for each meta-analysis.	4
